# Rubicon-Dependent Lc3 Recruitment to *Salmonella*-Containing Phagosomes Is a Host Defense Mechanism Triggered Independently From Major Bacterial Virulence Factors

**DOI:** 10.3389/fcimb.2019.00279

**Published:** 2019-08-02

**Authors:** Samrah Masud, Lars van der Burg, Lisanne Storm, Tomasz K. Prajsnar, Annemarie H. Meijer

**Affiliations:** Institute of Biology Leiden, Leiden University, Leiden, Netherlands

**Keywords:** Rubicon, Lc3-associated phagocytosis, *Salmonella*, autophagy, virulence factors

## Abstract

Intracellular pathogens such as *Salmonella* depend on their molecular virulence factors to evade host defense responses like autophagy. Using a zebrafish systemic infection model, we have previously shown that phagocytes, predominantly macrophages, target *Salmonella* Typhimurium by an autophagy-related pathway known as Lc3-associated phagocytosis (LAP), which is dependent on the host protein Rubicon. Here, we explore the influence of *Salmonella* virulence factors on pathogenicity in the zebrafish model and induction of LAP as a defense response. We investigated five mutant strains that all could trigger GFP-Lc3 recruitment as puncta or rings around single bacteria or bacterial clusters, in a Rubicon-dependent manner. We found that *S*. Typhimurium strains carrying mutations in PhoP or PurA, responsible for adaptation to the intracellular environment and efficient metabolism of purines, respectively, are attenuated in the zebrafish model. However, both strains show increased virulence when LAP is inhibited by knockdown of Rubicon. Mutations in type III secretion systems 1 and 2, SipB and SsrB, which are important for invading and replicating in non-phagocytic cells, did not affect the ability to establish successful infection in the zebrafish model. This observation is in line with our previous characterization of this infection model revealing that macrophages actively phagocytose the majority of *S*. Typhimurium. In contrast to SipB mutants, SsrB mutants were unable to become more virulent in Rubicon-deficient hosts, suggesting that type III system 2 effectors are important for intracellular replication of *Salmonella* in the absence of LAP. Finally, we found that mutation of FlhD, required for production of flagella, renders *S*. Typhimurium hypervirulent both in wild type zebrafish embryos and in Rubicon-deficient hosts. FlhD mutation also led to lower levels of GFP-Lc3 recruitment compared with the wild type strain, indicating that recognition of flagellin by the host innate immune system promotes the LAP response. Together, our results provide new evidence that the Rubicon-dependent LAP process is an important defense mechanism against *S*. Typhimurium.

## Introduction

*Salmonella enterica* serovar Typhimurium (*S*. Typhimurium) is one of the most common causes of foodborne gastroenteritis in humans, claiming more than 150,000 lives each year (Majowicz et al., 2010). Although *S*. Typhimurium infections generally resolve without treatment, immunocompromised patients can develop serious complications, such as bacteremia and other forms of systemic infection. *S*. Typhimurium also causes a systemic infection in mice that resembles typhoid fever, as caused in humans by another *S*. enterica serovar, *S*. Typhi. *S*. Typhimurium and *S*. Typhi are facultative intracellular pathogens that are able to replicate in both phagocytic and non-phagocytic cells. To this end, these pathogens employ a broad range of virulence strategies that mediate host cell invasion, growth in the intracellular environment, and subversion of the host cell's microbicidal mechanisms. These virulence strategies depend for a major part on effector proteins translocated by two type III secretion systems (T3SSs), T3SS1 and T3SS2, encoded by *Salmonella* pathogenicity island (SPI), SPI1 and SPI2, respectively (Ibarra and Steele-Mortimer, 2009). Other factors, such as flagellar motility and the ability to make structural and metabolic adaptations to its environment, also play important roles in *Salmonella* virulence.

*S*. Typhimurium accomplishes active invasion into non-phagocytic cells via the T3SS1 secretion system, which translocates a limited number of tightly regulated effector proteins (Myeni et al., 2013) that collectively induce dramatic changes to the host cell cytoskeleton leading to membrane ruffling and ultimately resulting in bacterial internalization (McGhie et al., 2009). The delivery of these SPI1 effectors is dependent on translocator proteins like SipB and SipC, which are also encoded in the SPI1 region (Myeni et al., 2013). Mutation of SipB has been shown to attenuate adhesion and invasion of HeLa cells as well as virulence and persistence of *S*. Typhimurium in mice (Chen et al., 2015). In contrast, professional phagocytes such as macrophages recognize *S*. Typhimurium-related pathogen associated molecular patterns (PAMPs) via pattern recognition receptors (PRRs), and utilize their phagocytic abilities for direct uptake of the bacterial cells. This recognition and phagocytosis initiates a number of host signaling responses in phagocytes including a bactericidal oxidative burst, cytokine production, and the activation of autophagy (Huang et al., 2009; Levine et al., 2011; Dupré-Crochet et al., 2013).

Once internalized, *S*. Typhimurium survives by avoiding anti-bacterial host responses and replicates intracellularly inside a modified phagosome, the *Salmonella*-containing vacuole (SCV). The function of the T3SS2 secretion system, translocating SPI2 effector proteins such as SifA, SseF, and SseG, is important for the maturation and maintenance of this intracellular niche (Hensel et al., 1998; Kuhle and Hensel, 2002). Expression of the T3SS2 is activated by the two-component system SsrA-SsrB (Walthers et al., 2007). SsrB has been shown to activate a regulon of genes located not only within but also outside the SPI2 locus, and also represses the expression of SPI1 genes during the intracellular stages of infection (Worley et al., 2000; Pérez-Morales et al., 2017). Other factors that play a role in *S*. Typhimurium pathogenesis include flagellar-based motility, which increases invasiveness of *Salmonella* (Schmitt et al., 2001). On the other hand, recognition of flagellin by host PPRs induces the innate immune response (Franchi et al., 2006; Miao et al., 2006). *S*. Typhimurium highly depends on a number of metabolic products to survive intracellularly and cause invasiveness. One such factor is PurA (succinyl AMP synthase), which functions in purine metabolism by converting IMP to AMP (Benson and Gots, 1976; McFarland and Stocker, 1987). PurA mutants grow poorly *in vivo* and are non-virulent in mice (O'Callaghan et al., 1988). Additionally, *S*. Typhimurium is highly dependent on the PhoP/PhoQ two-component system to survive inside professional phagocytes (Miller et al., 1989; Bijlsma and Groisman, 2005). The PhoP/PhoQ system detects acidification of the phagosome and presence of cationic antimicrobial peptides, leading to essential adaptations of the protein and lipid contents of the outer membrane (Dalebroux and Miller, 2014).

Recently, autophagy has emerged as an important host defense mechanism against *S*. Typhimurium and other intracellular pathogens. Autophagy is a cellular degradative pathway that can target bacterial invaders in a similar manner as it destructs defective organelles or protein aggregates (Levine et al., 2011; Huang and Brumell, 2014). A number of studies in epithelial cells have shown that *Salmonella* bacteria escaping from the SCV into the cytosol are ubiquitinated, recognized by ubiquitin receptors, enclosed in a double membrane autophagosome, and degraded following the fusion with lysosomes (Birmingham et al., 2006; Huang et al., 2009; Thurston et al., 2009, 2012, 2016; Zheng et al., 2009; Cemma et al., 2011; Wild et al., 2011). This selective, ubiquitin-dependent, autophagy mechanism, can also detect damage to the SCV membrane, likely functioning as a repair mechanism to delay cytosolic escape (Thurston et al., 2012). Alternatively, components of the autophagy machinery can be recruited directly to phagosomes, in a process that has been termed Lc3-associated phagocytosis (LAP) (Sanjuan et al., 2007; Huang and Brumell, 2014). LAP is initiated by PRRs, including Toll-like receptors (TLRs), and characterized by recruitment of the autophagy component LC3 to the phagosomal membrane and subsequent phagosome fusion with lysosomes, leading to rapid acidification and enhanced bacterial killing (Sanjuan et al., 2007).

A key player in LAP is RUN and cysteine rich domain containing BECLIN1 interacting protein (Rubicon). Rubicon is found to regulate autophagy by modulating the activity of the PI3 kinase Vps34 (Zhong et al., 2009). Under normal conditions, Rubicon suppresses autophagy by interacting with the Beclin-1-Vps34 complex (Matsunaga et al., 2009). However, upon TLR stimulation, Rubicon interacts with the NADPH oxidase complex on phagosomes. By recruiting the p40^phox^ component (NCF4) and subsequently stabilizing the p22^phox^ (CYBA) component of the NADPH oxidase, Rubicon activity induces the production of bactericidal reactive oxygen species (ROS) (Yang et al., 2012; Martinez et al., 2015). Thus, Rubicon functions as a molecular switch between canonical autophagy and LAP.

We have recently shown that both Rubicon and NADPH oxidase are required for Lc3 recruitment to *S*. Typhimurium in a systemically challenged zebrafish host (Masud et al., 2019). In this infection model, *Salmonella* bacteria, which are intravenously delivered into zebrafish embryos, primarily infect macrophages, but also neutrophils, and eventually continue to grow extracellularly in the bloodstream and cause a lethal infection (van der Sar et al., 2003). Deficiency of either Rubicon or NADPH oxidase not only reduced Lc3-*Salmonella* associations but also increased the mortality rate. While canonical autophagy may target *Salmonella* at later stages of infection, we found that the initial Lc3 response to *Salmonella* is independent of the autophagy pre-initiation factor Atg13, which led us to conclude that LAP is the major autophagic pathway involved in the anti-*Salmonella* reaction under conditions where macrophages dominate the defense response (Masud et al., 2019). In the present study we investigated which of the *S*. Typhimurium virulence factors, PhoP, PurA, SipB, SsrB, and FlhD, are required for pathogenicity in this systemic infection model, and how these factors affect the LAP response. Our study revealed that PhoP and PurA facilitate *S*. Typhimurium replication in the zebrafish host, while FlhD-regulated expression of the flagellar apparatus benefits host defense. We found LAP to occur as a general response to *S*. Typhimurium internalization, since Rubicon-dependent recruitment of Lc3 was observed in infections with both wild type and mutant strains. With the exception of Δ*ssrB* mutants, all tested strains displayed increased virulence under conditions of Rubicon knockdown, indicating that LAP functions to restrict *S*. Typhimurium growth and suggesting that SPI2 effectors promote *S*. Typhimurium virulence in the absence of LAP.

## Materials and Methods

### Zebrafish Lines and Maintenance

Zebrafish were handled in compliance with local animal welfare regulations and maintained according to standard protocols (zfin.org). Breeding of zebrafish was approved by the local animal welfare committee of Leiden University, under license number 10612. All experiments were performed on embryos/larvae before the free feeding stage and did not fall under animal experimentation law in line with the EU Animal Protection Directive 2010/63/EU. Fish lines used for the present work were the wild type (wt) strain AB/TL, and the transgenic line *Tg(CMV:GFP-map1lc3b)* (He et al., 2009). Embryos from adult fish were attained by natural spawning and were kept at 28.5°C in egg water (60 μg/ml sea salt, Sera Marin, Heinsberg, Germany). PTU (1-phenyl-2thiourea; Sigma Aldrich) was added to egg water at 0.003% to prevent melanization of embryos. For infection delivery and live imaging experiments embryos were anesthetized in egg water with 0.02% of buffered Tricane (3-aminobanzoic acid ethyl ester; Sigma Aldrich).

### Bacterial Cultures and Infection Experiments

*Salmonella* Typhimurium strains used in this study are listed in Table 1 along with their respective mutations and their description. The bacterial strains were plated fresh from −80°C stocks over LB agar plates with respective selection markers and were incubated overnight to grow at 37°C. Before the start of infection experiments, colonies from LB agar plates were suspended in phosphate buffered saline (PBS) supplemented with 2% polyvinylpyrrolidone-40 (Sigma Aldrich) to obtain the low dose (200–400 CFU, for survival curves and CFU counts experiments) or the high dose (2,000–4,000 CFU, for imaging experiments), as previously described (Masud et al., 2019). Bacterial inoculum was injected systemically into the caudal vein of the anesthetized embryos at 2 dpf. To check the inoculum size, the same dose was spotted onto agar plates, and bacterial counts determined following overnight incubation. After infection, embryos were kept individually in egg water in 48-well plates to score survival during larval development up to 5 dpf and to collect individuals for CFU counts at 24 h intervals.

**Table 1:** List of *Salmonella* strains used for study.

**Serial no**.	**Name of strain^**a**^**	**Mutation**
1	SL1344	None (wild type)
2	Δ*phoP*	Mutation in PhoP/Q two-component sensor of the host intracellular environment
3	Δ*ssrB*	Mutation in SsrB regulator of SPI2-proteins
4	Δ*flhD*	Mutation in flagellar transcription regulator
5	Δ*sipB*	Mutation in SPI1 translocator protein SipB
6	Δ*purA*	Mutation in adenylosuccinate synthetase (purine metabolism)

a *All strains were provided by Dirk Bumann (University of Basel). All mutant strains are in SL1344 background*.

### Determination of *in vivo* Bacterial (CFU) Counts

Five larvae per time point were sacrificed and homogenized in PBS using the Bullet Blender Tissue Homogenizer (Next Advance Inc.). Homogenates were then serially diluted, and three technical replicates for each embryo/larva were plated on LB solid media with the appropriate antibiotics for *S*. Typhimurium. To determine the CFUs, the resulting colonies were counted manually after 24 h incubation at 37°C.

### Morpholino Knockdown

Morpholino oligonucleotides (Gene Tools) used for Rubicon (*rubcn*) knockdown and control were diluted in Danieau buffer (58 mM NaCl, 0.7 mM KCl, 0.4 mM MgSO_4_, 0.6 mM Ca(NO_3_)_2_, 5.0 mM HEPES; pH 7.6) to obtain the required concentrations. *rubcn* knockdown was achieved by injecting 1 nl volume of 0. 25 mM of a previously described splice blocking morpholino (MO2-rubcn, CGCTGTGAAATCTGCTGACCTGAGC) (Masud et al., 2019). The knockdown effect was verified by RT-PCR with a pair of primers flanking the e6i6 boundary, Forward: TCTTATCAGCGCAGCTCAAAC and Reverse: GTGAAAATGGACCACAGCTCTT). Similarly, 1 nl of standard control morpholino was injected into the yolk of 0 hpf zebrafish embryos with microneedles and a Femtojet injector (Eppendorf) paired with a stereo-microscope.

### Imaging and Image Analysis

Confocal laser scanning images were acquired using at 63x water immersion objective (NA 1.2) with a Leica TCS SPE system, Samples were fixed at 4 hpi in 4% PFA and washed with PBS before image acquisitions. For Lc3-*Salmonella* associations, images acquired were analyzed through Z-stacks in Leica LAS AF Lite software and bacterial clusters were observed and manually counted in the overlay channel. Max projections in the overlay channels were used for representative images. For quantification of the autophagic response within infected phagocytes, for each embryo, the total number of observable phagocytes were manually counted through the Z-stacks of the acquired confocal image. Phagocytes were identified in the yolk sac circulation valley by bacterial clusters in the *mCherry* channel and cellular boundaries of phagocytes were determined in the light transmission channel. Among these total observable infected phagocytes, the numbers of cells with GFP-Lc3 signal in association with *Salmonella* bacteria were counted and the percentage of Lc3-positive phagocytes over the total observable phagocytes was determined for each embryo.

### Statistical Analyses

All data sets were analyzed with Prism 7 software. Survival curves were analyzed with Log rank (Mantel-Cox) test. For CFU counts, one way ANOVA was performed on Log transformed data and was corrected for multiple comparisons using Sidak's multiple comparisons test when required. Percentage Lc3-positive phagocyte quantifications was analyzed for significance with unpaired parametric *t*-test between two groups, and for multiple groups the one way ANOVA test was performed and corrected for multiple comparisons.

## Results

### Virulence of *S*. Typhimurium in the Zebrafish Model Relies on PhoP and PurA Factors

**Figure 1:**
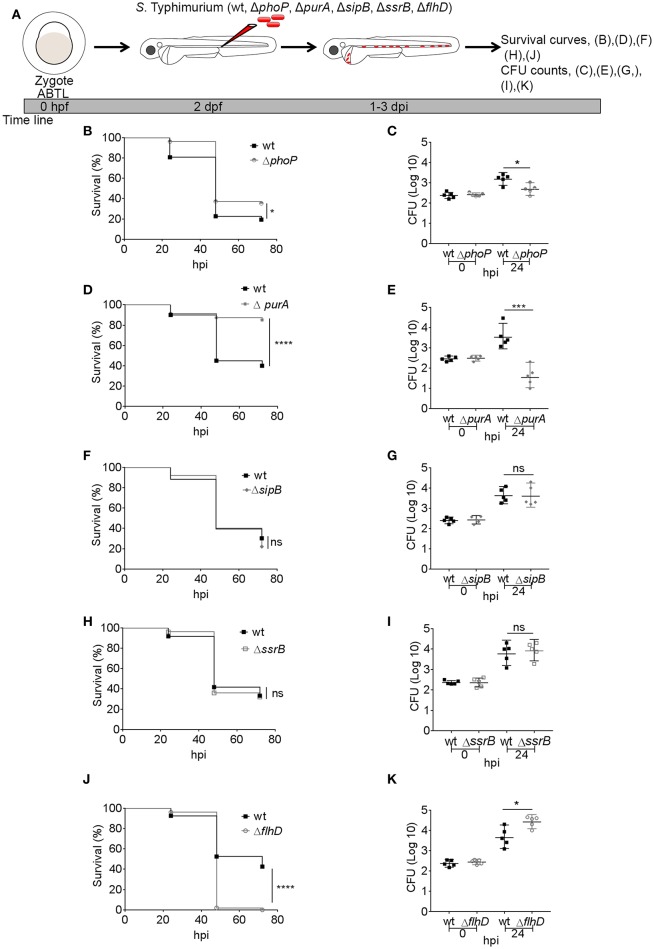
Comparison of virulence and infection progression of *S*. Typhimurium mutants. **(A)** Work flow for experiments followed in **(B–K)**, displayed along the time line of zebrafish development. **(B,D,F,H,J)** Survival curves for systemically challenged zebrafish with *S*. Typhimurium mutant strains, Δ*phoP* **(B)**, Δp*urA* **(D)**, Δ*sipB* **(F)**, Δ*ssrB* **(H)**, and Δ*flhD* **(J)** with their respective control groups injected with wild type strain SL1344. One representative of three replicates is shown (*n* = 50 embryos per group). **(C,E,G,I,K)** Representative CFU counts of the infections with *S*. Typhimurium mutant strains, Δ*phoP* **(C)**, Δ*purA* **(E)**, Δ*sipB* **(G)**, Δ*ssrB* **(I)**, and Δ*flhD* **(K)** with their respective control groups injected with wild type strain SL1344 at 24 hpi. One representative of three replicates is shown, where five embryos per time point were used and the log transformed CFU data are shown with the geometric mean per time point. ^****^P < 0.0001, ^***^P < 0.001, ^*^P < 0.05, ns, non-significant.

*S*. Typhimurium requires a number of effector proteins for infecting a wide range of cell types, including macrophages (Buckner and Finlay, 2011; LaRock et al., 2015). Mutant strains of *S*. Typhimurium that cannot survive inside macrophages are avirulent (Fields et al., 1986), but the interaction of the macrophage defense machinery with *S*. Typhimurium is not well-understood (Gog et al., 2012). PhoP, which is part of the PhoP/Q two component regulatory system, has been shown to be an essential virulence factor as bacteria containing an inactive PhoP are defective in intracellular survival in murine macrophages (Miller et al., 1989). We previously showed that *S*. Typhimurium infection in zebrafish embryos/larvae predominantly resides inside macrophages (Masud et al., 2019), providing an *in vivo* context to determine the importance of PhoP for establishing systemic infection. To this end, we challenged 2 days post fertilization (2 dpf) embryos either with a PhoP-deficient *S*. Typhimurium mutant strain or its wild type counterpart (SL1344) (Figure 1A). We evaluated the relative virulence of both strains on the basis of survival of infected embryos following intravenous injection of 200–400 colony forming units (CFU) and monitored bacterial burdens in infected hosts. We observed that Δ*phoP*-infected larvae showed a minor but significant increase in survival rates as compared to individuals infected with the wild type strain (Figure 1B). Similarly, the retrieved bacterial counts for hosts infected with the Δ*phoP* strain were significantly lower at 24 h post infection (24 hpi) as compared to wild type bacteria (Figure 1C). These results confirm that PhoP contributes to *S*. Typhimurium infection establishment and virulence in the zebrafish host.

Another important factor for S. Typhimurium virulence *in vitro* (Grant et al., 2012) and *in vivo* models (Benson and Gots, 1976; O'Callaghan et al., 1988) is PurA, which is required by *S*. Typhimurium for metabolic adaptation to the host environment (McFarland and Stocker, 1987). We found that a PurA-deficient *S*. Typhimurium (Δ*purA*) strain was avirulent to zebrafish larvae and failed to cause mortalities in comparison to the wild type strain (Figure 1D). Similarly, the Δ*purA* strain could not establish a successful infection as depicted by significantly lower numbers of CFU counts at 24 hpi in Δ*purA* infected larvae when compared to larvae infected with the wild type strain (Figure 1E).

### SPI1 and SPI2 Factors, SipB, and SsrB Are Dispensible for Establishing Systemic Infection in Zebrafish Embryos

*S*. Typhimurium depends on active invasion and formation of the SCV to successfully infect and survive inside non-phagocytic host cells (LaRock et al., 2015). However, in our model, active invasion by *S*. Typhimurium is not required due to swift phagocytosis of the bacteria by phagocytes, predominantly macrophages but also neutrophils, following injection into the bloodstream of the zebrafish host (Masud et al., 2019). We therefore investigated the hypothesis that SPI1 effectors, required for active invasion, would be dispensable for virulence in this model. To this end, we infected zebrafish embryos with a SipB-deficient *S*. Typhimurium strain, which fails to produce the translocation apparatus for delivery of SPI1 effectors, resulting in severe inability to cause infections in non-phagocytic cells (Kaniga et al., 1995; Myeni et al., 2013). The Δ*sipB* strain and its isogenic wild type strain caused similar mortalities to zebrafish larvae (Figure 1F). Moreover, the *sipB* mutant *S*. Typhimurium was equally virulent as the wild type strain, as CFU counts at 24 hpi for Δ*sipB* and wild type did not differ from each other (Figure 1G).

In order to determine the requirement for SPI2 effectors in our model, we used an SsrB-deficient strain. SsrB is a part of the two component regulatory system SsrA/B and is required for expression of most of the SPI2 proteins, required for maintenance of the SCV (Walthers et al., 2007). We found no significant difference in survival rates (Figure 1H) and in CFU counts (Figure 1I) between the groups injected with Δ*ssrB* or wild type *S*. Typhimurium strains. Collectively, our results suggest that both SipB and SsrB are less relevant factors for pathogenicity of *S*. Typhimurium in the zebrafish-*Salmonella* infection model, presumably due to no or little requirement of active bacterial invasion into phagocytes.

### Mutation of the Flagellin Transcriptional Regulator FlhD Increases Virulence of *S*. Typhimurium in the Systemically Infected Zebrafish Host

FlhD is a part of the *flhDC* master operon responsible for initiating the flagellum production. Mutations in FlhD factor render *S*. Typhimurium immotile due to loss of flagellin (a main component of the bacterial flagellum) synthesis. Flagellin positively and negatively affects *Salmonella* virulence as *Salmonella* requires flagellum-based motility for optimal invasion of the host cells (Ibarra and Steele-Mortimer, 2009), but on the other hand flagellin is a ligand for TLR pathway detection by the host, activating the innate immune response (Hayashi et al., 2001). We have previously shown that injection of *Salmonella* flagellin into zebrafish embryos induces innate immune response genes (e.g., *il1b*) and that this response is reduced under knockdown conditions of the flagellin receptors, Tlr5a/b, or by mutation of Myd88, which functions downstream of these receptors (Stockhammer et al., 2009; van der Vaart et al., 2013). As *Salmonella* is rapidly phagocytosed by macrophages and neutrophils in our intravenous infection model (Masud et al., 2019), we hypothesized that the Δ*flhD* mutation would have little impact on the ability of *Salmonella* for host invasion, but would favor bacterial growth due to evasion of innate immune detection. In order to test this hypothesis, we infected zebrafish larvae with the Δ*flhD* strain or its wild type counterpart and determined the virulence in terms of CFU counts and survival curves, as above. As expected, infection with the Δ*flhD* strain led to reduced host survival (Figure 1J) and increased bacterial proliferation (Figure 1K) when compared to infection with the wild type strain. These results show that the presence of a flagellum helps the zebrafish host to restrict *S*. Typhimurium growth in the intravenous infection model.

### *S*. Typhimurium Virulence Factor Mutants Elicit GFP-Lc3 Recruitment in Similar Patterns as the Wild Type Strain

**Figure 2:**
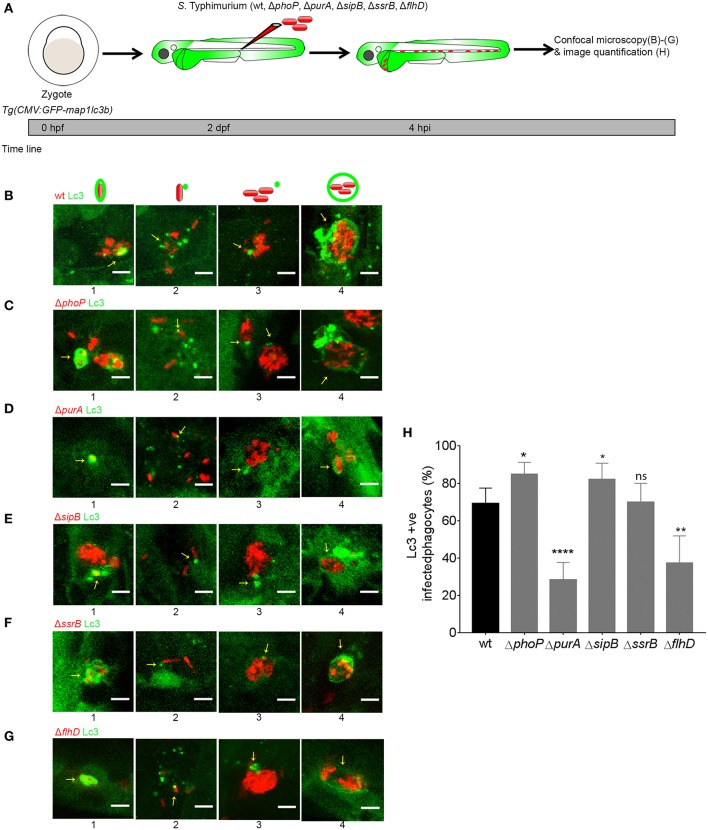
Host LAP response to *S*. Typhimurium mutants. **(A)** Work flow and time line of experiments followed in **(B–H)**. **(B–G)** Representative confocal micrographs of GFP-Lc3 positive infected phagocytes with *mCherry*-labeled *S*. Typhimurium mutant strains wild type **(B)**, Δ*phoP* **(C)**, Δ*purA* **(D)**, Δ*sipB* **(E)**, Δ*ssrB* **(F)**, and Δ*flhD* **(G)**. Yellow arrows indicate GFP-Lc3 associations with bacterial cells and numbers 1–4 represent four different examples of Lc3 association patterns for each *S*. Typhimurium mutant strain. At the top of each column of images is a symbolic representation of the pattern of GFP-Lc3 and bacterial cell association as reported earlier (Masud et al., 2019). **(H)** Quantification of GFP-Lc3-*Salmonella* association for *S*. Typhimurium mutant strains Δ*phoP*, Δ*purA*, Δ*sipB*, Δ*ssrB*, and Δ*flhD* along with wild type SL1344 at 4 hpi. Numbers of infected phagocytes positive or negative for GFP-Lc3-*Salmonella* associations were counted from confocal images and the percentages of Lc3-positive (Lc3+ve) over the total were averaged from five embryos per group. Error bars represent the SD. One of the two replicates is shown. Scale bar **(B–G)** = 5 μm, ^****^P < 0.0001, ^**^P < 0.01, ^*^P < 0.05, ns, non-significant.

We recently identified a host protective role for the autophagy-related mechanism known as Lc3-associated phagocytosis (LAP) during infection of zebrafish embryos with *S*. Typhimurium (Masud et al., 2019). In the present work, we decided to determine whether LAP also targets *S*. Typhimurium with non-functional PhoP, PurA, SipB, SsrB, and FlhD factors. To visualize this host defense response we injected a high dose (2,000–4,000 CFU) of each bacterial mutant strain or the wild type strain into the *Tg(CMV:GFP-map1lc3b)* transgenic zebrafish line, which expresses a GFP-fusion of the autophagy marker Lc3 (Figure 2A). We investigated GFP-Lc3-*Salmonella* associations at 4 hpi, where the LAP response is highest based on previous time course analysis (Masud et al., 2019). We could observe GFP-Lc3 associations with all *S*. Typhimurium virulence mutants tested (Figures 2B–G). Furthermore, the GFP-Lc3 localization patterns observed in response to infections with the mutant *S*. Typhimurium strains were similar to those observed with the wild type strain (Masud et al., 2019; Figures 2B1–4), as in all cases the GFP-Lc3 signal appeared either in the form of fluorescent puncta associated with single bacterial cells or bacterial clusters, or as fluorescent rings around the bacterial cells or clusters (Figures 2B–G). However, differences were observed in the total level of GFP-Lc3 recruitment between the strains (Figure 2H). Infection with Δ*phoP* and Δ*SipB* strains elicited slightly higher levels of GFP-Lc3 recruitment, whereas GFP-Lc3 recruitment was reduced in infections with the Δ*FlhD* and Δ*purA* strains (Figure 2H). These differences in GFP-Lc3 recruitment did not correlate with the virulence of the mutants, since Δ*phoP* and Δ*purA* were attenuated and Δ*flhD* was more virulent in the zebrafish model (Figure 1).

### *S*. Typhimurium Virulence Factor Mutants Are Targeted by the Rubicon-Dependent LAP Pathway

**Figure 3:**
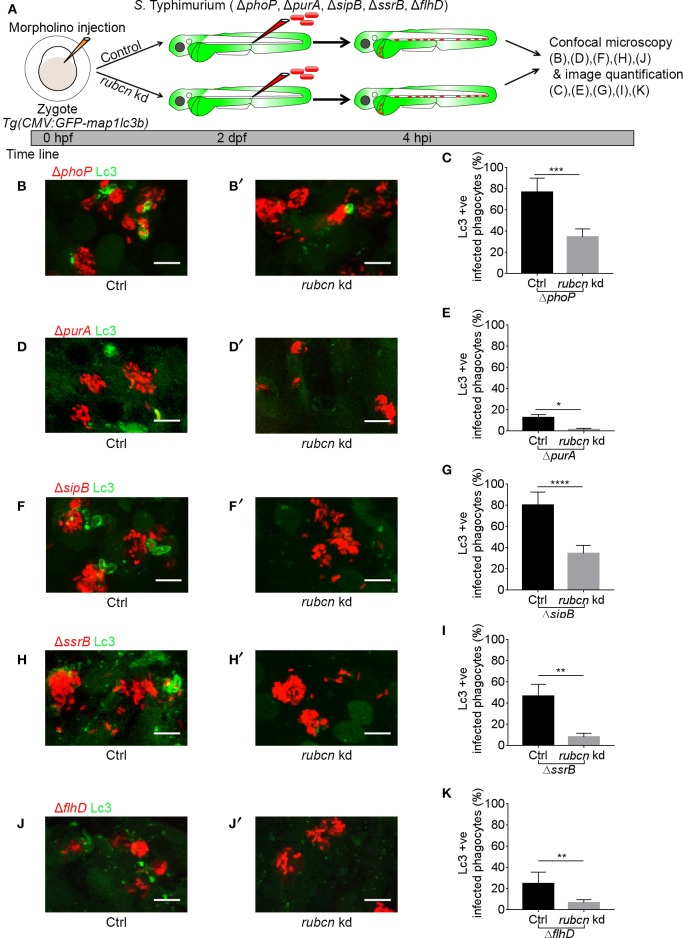
Host LAP response to *S*. Typhimurium mutants in Rubicon-depleted embryos. **(A)** Work flow and time line of experiments in **(B–K)**. **(B,D,F,H,J)** Representative confocal micrographs of GFP-Lc3 positive infected phagocytes with *mCherry*-labeled *S*. Typhimurium mutant strains Δ*phoP* **(B)**, Δ*purA* **(D)**, Δ*sipB* **(F)**, Δ*ssrB* **(H)**, and Δ*flhD* **(J)** in Rubicon-depleted hosts **(B',D',F',H',J')** along with respective controls **(B,D,F,H,J)**. **(C,E,G,I,K)** Quantification of GFP-Lc3-*Salmonella* association for *S*. Typhimurium mutant strains Δ*phoP* **(C)**, Δ*purA* **(E)**, Δ*sipB* **(G)**, Δ*ssrB* **(I)**, and Δ*flhD* **(K)** in Rubicon-depleted larvae and their respective controls. Numbers of infected phagocytes positive or negative for GFP-Lc3-*Salmonella* associations were counted from confocal images and the percentages of Lc3-positive over the total were averaged from five embryos per group. Error bars represent the SD. One of the two replicates is shown. Scale bar **(B,D,F,H,J)** = 10 μm, ^****^P < 0.0001, ^***^P < 0.001, ^**^P < 0.01, ^*^P < 0.05.

In order to determine if the GFP-Lc3 response to the different *S*. Typhimurium mutant strains can be classified as LAP, we tested for dependency on the host protein Rubicon, which is essential for LAP but inhibits canonical autophagy (Martinez et al., 2015). To deplete Rubicon, we used a previously described splice blocking morpholino oligonucleotide (Masud et al., 2019) and validated the knockdown effect by RT-PCR (Supplementary Figure 1). Rubicon depletion resulted in significantly reduced levels of GFP-Lc3 recruitment in response to the Δ*phoP*, Δ*purA*, Δ*sipB* Δ*ssrB*, and Δ*flhD* strains (Figure 3), in agreement with previous evidence for the role of LAP during infection with wild type *S*. Typhimurium (Masud et al., 2019). In case of the Δ*purA*, Δ*ssrB*, and Δ*flhD* mutants, GFP-Lc3 association was almost completely abolished under conditions of Rubicon knockdown (Figures 3D,E,H–K). In contrast, during infections with the Δ*phoP* and Δ*sipB* strains, Rubicon-deficient hosts displayed residual levels of GFP-Lc3-*Salmonella* association (Figures 3B,C,F,G), suggesting that canonical autophagy might target these strains when LAP is inhibited. Together, these results extend our previous findings and strengthen our conclusion that LAP is the main autophagy-related response of phagocytes, predominantly macrophages, targeting *S*. Typhimurium during systemic infection in zebrafish larvae.

### *S*. Typhimurium Mutants Show Increased Virulence in a LAP-Deficient Host With the Exception of Δ*ssrB*

**Figure 4:**
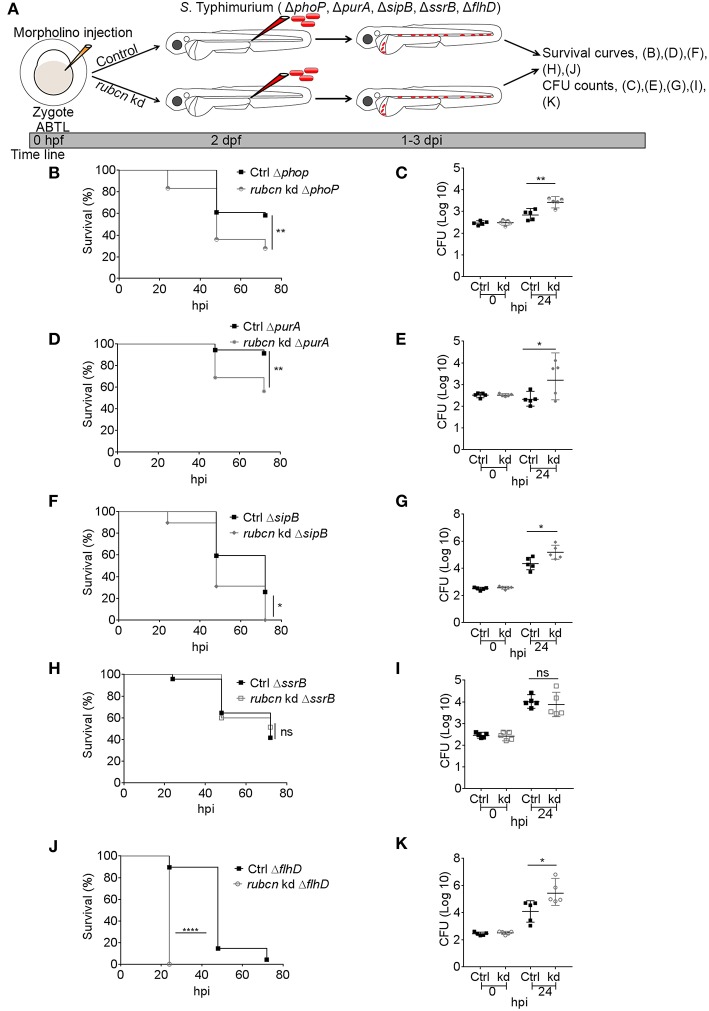
Virulence and infection progression of *S*. Typhimurium mutants in Rubicon-depleted hosts. **(A)** Work flow and time line of experiments followed in **(B–K)**. **(B,D,F,H,J)** Survival curves for systemically injected *S*. Typhimurium mutant strains, Δ*phoP* **(B)**, Δ*purA* **(D)**, Δ*sipB* **(F)**, Δ*ssrB* **(H)**, and Δ*flhD* **(J)** in Rubicon-depleted larvae with their respective control groups. One representative of two replicates is shown (*n* = 50 embryos per group). **(C,E,G,I,K)** Representative CFU counts of the infections with *S*. Typhimurium mutant strains, Δ*phoP* **(C)**, Δ*purA* **(E)**, Δ*sipB* **(G)**, Δ*ssrB* **(I)**, and Δ*flhD* **(K)** in Rubicon-depleted zebrafish with their respective control groups at 24 hpi. One representative of two replicates is shown, where five embryos per time point were used and the log transformed CFU data are shown with the geometric mean per time point. Error bars represent SD. ^****^P < 0.0001, ^**^P < 0.01, ^*^P < 0.05, ns, non-significant.

Our previous results showed that LAP-deficient zebrafish embryos are more susceptible to wild type *S*. Typhimurium infection (Masud et al., 2019). To build further on this finding, we investigated the importance of LAP in defense against the *S*. Typhimurium mutant strains. In order to inhibit LAP we knocked down host Rubicon with the splice blocking morpholino approach and assessed the effect on host survival and bacterial burden (Figure 4A). We found that infections with Δ*phoP*, Δ*purA*, Δ*sipB*, and Δ*flhD* (Figures 4B,D,F,J) resulted in significantly increased mortality of Rubicon-deficient hosts as compared to control embryos. In agreement, the CFU counts for these strains were significantly increased in Rubicon-deficient embryos (Figures 4C,E,G,K). In contrast, the virulence of the Δ*ssrB* strain and its infection establishment were independent of Rubicon levels of the host, as there were no significant differences between the survival curves of Rubicon-deficient and control larvae (Figure 4H) and the CFU counts (Figure 4I). Therefore, we conclude that the Rubicon-dependent LAP response is required to restrict growth of *S*. Typhimurium strains carrying mutations in critical virulence factors and that only a strain deficient in expression of SPI2 factors (Δ*ssrB*) is unable to display increased virulence in a LAP-deficient host.

## Discussion

The functions of *S*. Typhimurium virulence factors in invasion of host cells and intracellular replication are well-described as a result of many years of studies in various cell culture and mouse models (Ibarra and Steele-Mortimer, 2009; van der Heijden and Finlay, 2012). However, how these factors induce or inhibit the host autophagy machinery is only beginning to be understood (Mesquita et al., 2012; Tattoli et al., 2012a,b; Owen et al., 2014). Recently, we exploited GFP-Lc3 transgenic zebrafish to study the dynamics of autophagy activation *in vivo*. Following intravenous infection in zebrafish embryos, *S*. Typhimurium is mainly contained by macrophages and as such this model mimics the advanced stages of systemic infections in humans. We found that internalization of live *S*. Typhimurium by macrophages in the zebrafish host is associated with rapid induction of the autophagy-related response known as LAP, whereby Lc3 associates with phagosomes in a Rubicon and NADPH oxidase dependent manner and independent of the ULK1 pre-initiation complex (Masud et al., 2019). Here we provide new evidence for the host-protective function of LAP, showing that *S*. Typhimurium strains carrying mutations in virulence factors PhoP, PurA, SipB, SsrB, and FlhD are all able to trigger LAP and that all mutants except the SsrB-deficient strain become more virulent in a LAP-deficient host.

It has previously been shown that lipopolysaccharide mutants are less virulent than wild type *S*. Typhimurium in the zebrafish model (van der Sar et al., 2003). However, the functions of other critical virulence factors had not yet been well-characterized. In this study we show the importance of two virulence factors, PhoP and PurA, for *S*. Typhimurium infection in the zebrafish host. The PhoP/Q regulon, which controls outer membrane composition, has been reported to inhibit phagolysosomal fusion and also to enable adaptation to other intramacrophage stresses (Garvis et al., 2001; Thompson et al., 2011). In addition, it has been suggested to decrease immune responses, by reducing TLR activation (Dalebroux and Miller, 2014). Infection with a PhoP-deficient strain led to attenuated infection in the zebrafish model, which is in good agreement with studies in murine macrophages and *in vivo* infection in mice (Miller et al., 1989; Miller and Mekalanos, 1990; Thompson et al., 2011), and with the fact that PhoP-deficient *S*. Typhi has been found safe for human vaccination (Hohmann et al., 1996). We observed an even stronger attenuation in the case of the PurA-deficient strain, which was largely avirulent in the zebrafish host. This observation is consistent with avirulence of PurA-deficient *S*. Typhimurium *in vivo* in mice (O'Callaghan et al., 1988). These results support the validity of the zebrafish model for studying *S*. Typhimurium infection.

When LAP was inhibited by knockdown of Rubicon, both the PhoP-deficient and the PurA-deficient strains induced more severe infections in the zebrafish host. That the zebrafish host becomes more susceptible to attenuated strains in the absence of LAP extends our previous finding that LAP is a host-protective response against *S*. Typhimurium (Masud et al., 2019). While both attenuated strains triggered recruitment of GFP-Lc3 in a Rubicon-dependent manner, they had opposite effects on the level of GFP-Lc3 recruitment, which was increased in the infection with PhoP-deficient bacteria and reduced in response to PurA-deficient bacteria. Since we examined only a single time point, this difference might be due to different kinetics in the clearance of bacteria. We have previously found that no GFP-Lc3 response to heat-killed bacteria is present at this time point (Masud et al., 2019). Therefore, the lower levels of GFP-Lc3 in response to Δ*purA S*. Typhimurium could be due to rapid clearance of this largely avirulent strain and failure to induce signals for LAP. The higher levels of GFP-Lc3 targeting the Δ*phoP* mutant could point toward a role for PhoP in LAP evasion, which adds to its other reported functions in escaping host defense mechanisms (Miller and Mekalanos, 1990; Garvis et al., 2001; Thompson et al., 2011; Dalebroux and Miller, 2014). Therefore, the PhoP deficiency might enhance intraphagosomal killing of *S*. Typhimurium in zebrafish phagocytes possibly due to higher LAP activity.

SipB-deficient bacteria, defective in the translocation of SPI1 effectors, showed similar virulence as wild type *S*. Typhimurium in the zebrafish model. SPI1 effectors are essential for gaining access to non-phagocytic target cells such as those in the gut epithelium (Watson and Holden, 2010), but this invasion step is bypassed in the intravenous infection route used for infecting zebrafish embryos. Therefore, the non-attenuated phenotype of Δ*sipB* infection in zebrafish is consistent with studies in mice showing that virulence of SPI1 mutants is unaffected when the bacteria are delivered intraperitoneally, whilst administration by the oral route affects the ability to establish systemic infection (Galán and Curtiss, 1989). In macrophages, SPI1 effectors can trigger caspase-1 dependent pyroptosis and SPI1 mutants, including Δ*sipB*, lack the ability to induce this response (Fink and Cookson, 2007). We cannot exclude that SipB deficiency might affect macrophage or neutrophil cell death in the zebrafish host. When *Salmonella* bacteria are released from phagocytes, they can continue to replicate extracellularly by forming microcolonies on the endothelial cells of the blood vessels (van der Sar et al., 2003). Therefore, it is possible that SipB mutation could have altered the ratio between intra- and extracellular bacteria, without alteration in overall bacterial burden or effect on host survival. Our observation of a small but significant increase in GFP-Lc3 recruitment by Δ*sipB* bacteria compared with the wild strain suggests increased targeting of the mutant strain by LAP, but apparently insufficient to impact on the overall infection development. However, as in the case of Δ*phoP* and Δ*purA* mutants, depletion of Rubicon revealed the host-protective function of LAP during infection with the Δ*sipB* strain.

Mutation of SsrB suppresses expression of all SPI2 genes and several genes beyond the SPI2 locus (Worley et al., 2000). Loss of SsrB did not significantly affect GFP-Lc3 recruitment and had no detectable effect on virulence in the zebrafish host. In contrast, Δ*ssrB* Salmonella bacteria are attenuated in several *in vitro* and *in vivo* models, including human epithelial cells, murine and porcine macrophages, orally and intravenously infected mice, and intravenously infected pigs (Cirillo et al., 1998; Boyen et al., 2008; Grant et al., 2012). However, a study in mice reported that the main cause of the attenuated phenotype of SPI2 mutants is their inability to leave infected cells, whereas growth of SPI2 mutants inside phagocytes exceeds that of wild type *Salmonella* (Grant et al., 2012). Therefore, the lack of an attenuated phenotype of Δ*ssrB* Salmonella in zebrafish embryos might be explained by the fact that phagocytes are the main replication site in this model. Interestingly, a function for SsrB in the zebrafish model was revealed when we inhibited LAP. We found that Δ*ssrB* bacteria, unlike all other mutants tested, were unable to display increased virulence under conditions of Rubicon knockdown. That only SsrB-competent strains become more virulent in a Rubicon-deficient host might be an indication that SPI2 effectors or other virulence proteins controlled by SsrB promote *S*. Typhimurium virulence in the absence of LAP, and therefore the *ssrB* mutant is unable to take advantage of the depletion of the host resistance factor Rubicon. However, it remains to be elucidated which of the many genes of the SsrB regulon might be involved in this process.

We found that loss of the FlhD regulator, which deprives *S*. Typhimurium from the formation of flagella, leads to increased virulence in both wild type and Rubicon-deficient zebrafish hosts. These results are in good agreement with studies in mice showing that non-flagellated mutants of *Salmonella*, including Δ*flhD*, are equally or more virulent than the wild type strains, despite that these mutants lack the ability to invade epithelial cells (Lockman and Curtiss, 1990; Schmitt et al., 2001; Fournier et al., 2009). The hypervirulence of non-flagellated bacteria can be explained by escape from recognition by TLR5-MyD88 signaling, as has been shown in mice (Fournier et al., 2009). This hypothesis is supported by our previous results showing that Tlr5a/b and MyD88 are also required for activation of the innate immune response to flagellin in zebrafish (Stockhammer et al., 2009; van der Vaart et al., 2013). An additional explanation for hypervirulence of the Δ*flhD* strain is the escape from inflammasome activation and interleukin 1 beta secretion, activated by Nod-like receptor signaling (Franchi et al., 2006; Winter et al., 2009; Lai et al., 2013). In addition to being hypervirulent, the Δ*flhD* strain elicited significantly lower GFP-Lc3 recruitment than wild type bacteria. This observation is in line with the fact that LAP induction has been shown to depend on recognition of TLR ligands, although it should be noted that a direct link between flagellin-TLR5 signaling and LAP has not yet been reported (Sanjuan et al., 2007). Considering the evidence for a host-protective function of LAP provided by our study, we propose that the increased virulence of Δ*flhD S*. Typhimurium resides at least partly in lower activation of LAP through lack of flagellin detection by TLR5 or other pattern recognition receptors of the innate immune system.

In conclusion, we have demonstrated attenuated virulence of Δ*phoP* and Δ*purA S*. Typhimurium mutants in the zebrafish systemic infection model in contrast to hypervirulence of the non-flagellated Δ*flhD* mutant. Furthermore, our data support that the Rubicon-dependent LAP pathway plays an important role in host defense, since all tested mutant strains were able to cause more severe infections when LAP was inhibited, except for one strain deficient in the expression of SPI2 effectors. While LAP is the predominant autophagy-related response during the macrophage-dominated infection in zebrafish, selective autophagy mediated by ubiquitin receptors (xenophagy) has been found to restrict growth of *S*. Typhimurium in epithelial cells (Huang and Brumell, 2014). Therefore, there is accumulating evidence for the function of autophagy proteins in host defense against *S*. Typhimurium, encouraging further exploration of autophagy modulating drugs for host-directed therapy of antibiotic-resistant *Salmonella* infections.

## Data Availability

Datasets supporting the conclusions of the article, will be made available on request of any qualified researcher, without any undue delay.

## Ethics Statement

Zebrafish were handled in compliance with local animal welfare regulations and maintained according to standard protocols (zfin.org). Breeding of zebrafish was approved by the local animal welfare committee of Leiden University, under license number 10612. All experiments were performed on embryos/larvae before the free feeding stage and did not fall under animal experimentation law in line with the EU Animal Protection Directive 2010/63/EU.

## Author Contributions

SM, TP, and AM designed the study and wrote the manuscript. SM performed the experimental work and analyzed the data. LS and LvdB contributed to experiments. TP and AM supervised the study. All authors read and approved the final manuscript.

### Conflict of Interest Statement

The authors declare that the research was conducted in the absence of any commercial or financial relationships that could be construed as a potential conflict of interest.

## Supplementary Material

Supplementary Figure 1 RT-PCR verification of splice morpholino for MO2- rubcn. Electrophoresis gel scan of reverse transcription polymerase chain reaction (RT-PCR) products for control and *rubcn* knockdown groups used for determining splice morpholino (MO-2 rubcn) efficiency at several developmental stages up to 122 hpf. Arrowheads indicate correctly spliced products of control groups with the expected size of 738 bp. Arrows indicate incorrectly spliced products with intron 6 retention of *rubcn* knockdown groups confirming MO2-*rubcn* efficiency.
