# Supplementary figures and images for: Rubicon-Dependent Lc3 Recruitment to Salmonella-Containing Phagosomes Is a Host Defense Mechanism Triggered Independently From Major Bacterial Virulence Factors
Source: Front Cell Infect Microbiol. 2019 Aug 2;9:279. doi: 10.3389/fcimb.2019.00279 (PMC6688089; doi:10.3389/fcimb.2019.00279)

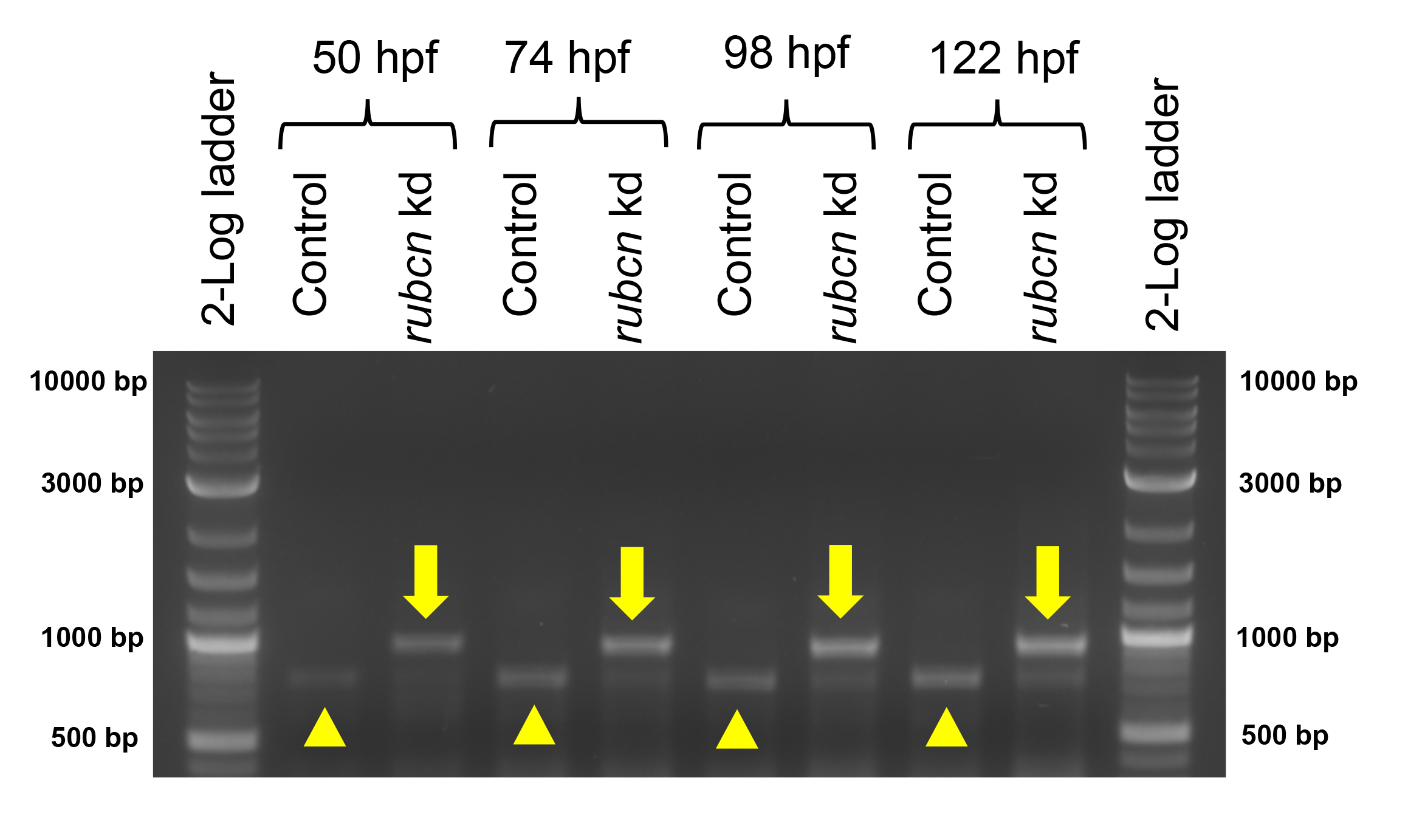

Supplement: Supplementary Figure 1 — RT-PCR verification of splice morpholino for MO2- rubcn. Electrophoresis gel scan of reverse transcription polymerase chain reaction (RT-PCR) products for control and rubcn knockdown groups used for determining splice morpholino (MO-2 rubcn) efficiency at several developmental stages up to 122 hpf. Arrowheads indicate correctly spliced products of control groups with the expected size of 738 bp. Arrows indicate incorrectly spliced products with intron 6 retention of rubcn knockdown groups confirming MO2-rubcn efficiency. [file Image_1.tiff]
